# An age-related decline in human fluid intelligence corresponds with immature astroglia prevalence

**DOI:** 10.3389/fncel.2026.1926804

**Published:** 2026-10-20

**Authors:** Aswathy Ammothumkandy, Luis Corona, Nadia A. Atai, Aidin Abedi, Nora Jimenez, Michelle Armacost, Lina M. D’Orazio, Virginia Zuverza-Chavarria, Alisha Cayce, Mohammad Shariq, Ryan McGinn, Darrin Lee, Brian Lee, Christianne Heck, Jonathan J. Russin, Charles Y. Liu, Jason A. D. Smith, Michael A. Bonaguidi

**Affiliations:** 1 Department of Stem Cell Biology and Regenerative Medicine, Eli and Edythe Broad Center for Regenerative Medicine and Stem Cell Research, Keck School of Medicine, University of Southern California, Los Angeles, CA, United States; 2 Neurorestoration Center, Keck School of Medicine, University of Southern California, Los Angeles, CA, United States; 3 Department of Neurological Surgery, Keck School of Medicine, University of Southern California, Los Angeles, CA, United States; 4 Los Angeles General Medical Center, Los Angeles, CA, United States; 5 Department of Neurology, Keck School of Medicine, University of Southern California, Los Angeles, CA, United States; 6 Department of Neurology, Rancho Los Amigos National Rehabilitation Center, Downey, CA, United States; 7 Department of Psychology, Rancho Los Amigos National Rehabilitation Center, Downey, CA, United States; 8 Department of Neurosurgery, Rancho Los Amigos National Rehabilitation Center, Downey, CA, United States; 9 Department of Neurology, Medical University of South Carolina, Charleston, SC, United States; 10 Department of Biomedical Engineering, Viterbi School of Engineering, University of Southern California, Los Angeles, CA, United States; 11 Department of Gerontology, University of Southern California, Los Angeles, CA, United States; 12 Zilkha Neurogenetic Institute, Keck School of Medicine, University of Southern California, Los Angeles, CA, United States

**Keywords:** adult neurogenesis, aging, astroglia, cognition, epilepsy, human hippocampus, intelligence

## Abstract

Age-related cognitive decline remains a daunting health challenge, and targeting neuronal cells alone has been insufficient to halt its progression. Astroglia are emerging as key players in cognitive function and dysfunction, yet their relevance to age-associated cognitive decline in humans remains unclear. Epilepsy patients undergo age-amplified progressive cognitive decline. Our prior research in Mesial Temporal Lobe Epilepsy (MTLE) patients identified pathological stellar Dcx + immature astroglia in epileptic, but not healthy hippocampus. In this study, we combined histology data with pre-surgical neuropsychology tests to assess (1) age-associated cognitive decline in MTLE patients (*N* = 40), (2) age-related changes in Dcx + immature astroglia (*N* = 29), and (3) the association between Dcx + immature astroglia and cognitive decline (*N* = 19). We observed a strong decline in fluid intelligence, visuospatial reconstruction, and memory with age. An increase in Dcx + immature astroglia positively correlated with age, whereas negatively associated with nonverbal abstract reasoning and fluid intelligence as measured by the Raven’s Progressive Matrices test. These findings reveal an interplay among aging, fluid intelligence decline, and immature astroglia prevalence in epilepsy patients. Our study suggests immature astroglia represent a new cellular target to consider for altering age-associated cognitive decline.

## Graphical abstract

**Figure:**
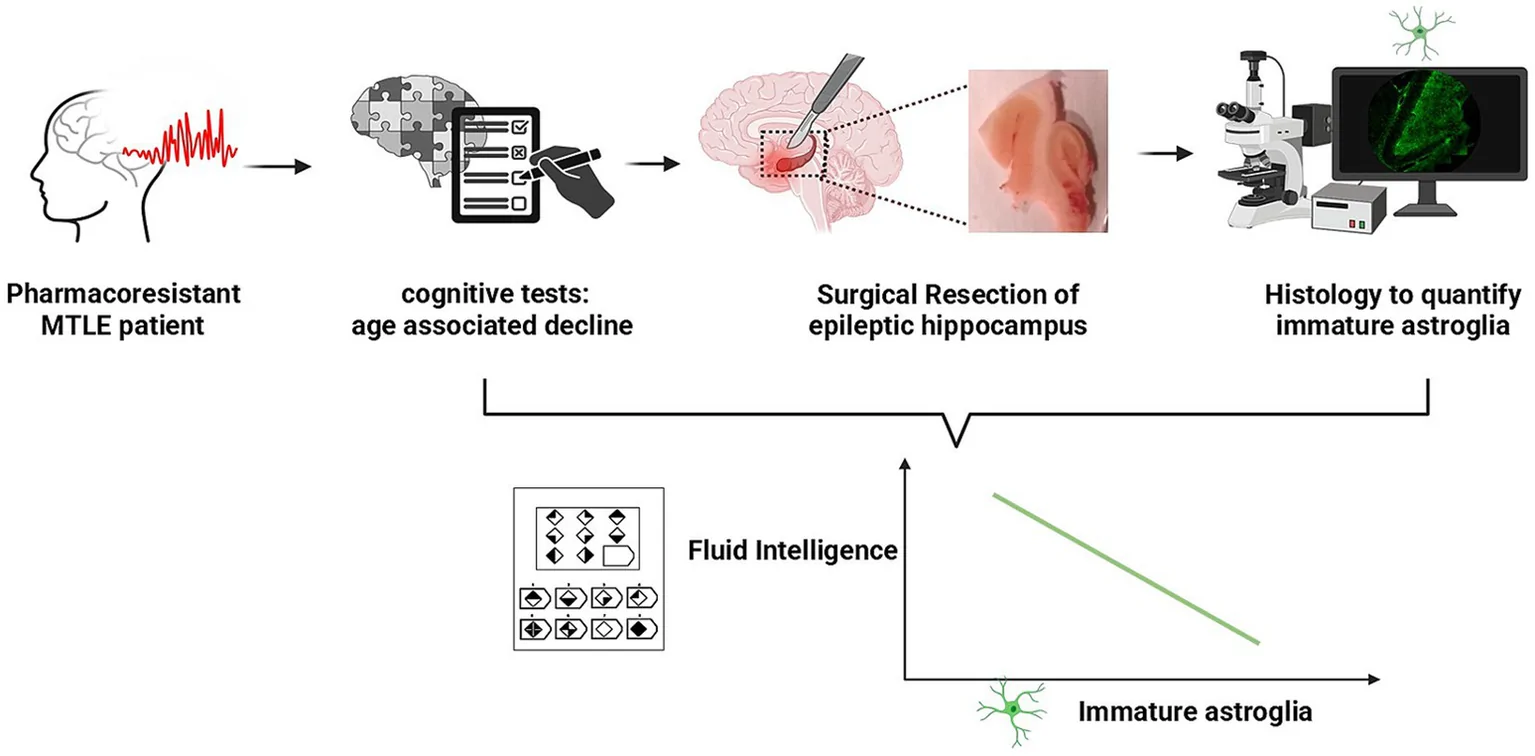

## Introduction

Cognitive decline due to aging and neurological disorders leads to poor quality of life, disability, loss of independence and imposes substantial socioeconomic costs (Feigin et al., 2019; Kesidou et al., 2023). Therapeutic development for cognitive decline has been steered by the interpretation that it predominantly results from compromised neuronal function, yet neuron-focused strategies have so far yielded only modest benefits. (Avila et al., 2017; Henstridge et al., 2019). With the advent of cell type–directed combination therapies targeting both neurons and glia (Li et al., 2025), identifying specific cellular drivers of discrete cognitive deficits can guide the design of more effective treatment strategies. Rodent studies have recognized that astroglial cells, traditionally viewed as mere supportive cells to neurons, are active regulators of learning and memory (Sardinha et al., 2017; Adamsky et al., 2018; Santello et al., 2019; Kol et al., 2020; Doron et al., 2022; Williamson et al., 2024; Dewa et al., 2025; Sánchez Romero and Navarrete, 2026). Meanwhile, various studies indicate that dysfunctional astrocytes contribute to the decline of rodent cognitive functions (Lee et al., 2014; Habbas et al., 2015; Orr et al., 2015; Santello et al., 2019; Licht-Murava et al., 2023). These cognitive studies in mice largely focus on spatial learning and memory, whereas human cognition encompasses additional domains such as intelligence, verbal learning and language that can have domain and species-specific cellular contributions (Ammothumkandy et al., 2025b). In people, elevated transcriptomic signatures of reactive astrocyte markers in brain and higher levels of markers in peripheral blood have been associated with greater global cognitive decline during aging and Alzheimer’s disease (Lye et al., 2019; Wu et al., 2023; Peretti et al., 2024; Ammothumkandy et al., 2025a). However, a direct cellular association between pathological astroglia and decline in specific cognitive domains remains unexplored in humans.

Cognitive decline is a comorbidity that occurs progressively in epilepsy patients. People with epilepsy demonstrate accelerated brain aging (Breuer et al., 2016; Hwang et al., 2020) and cognitive decline is especially pronounced in older individuals (Piazzini et al., 2006; Sen et al., 2018). Mesial Temporal Lobe Epilepsy (MTLE) is the most common form of adult epilepsy, and patients who do not respond to medications undergo surgical removal of epileptic hippocampal tissue as treatment (Engel, 2001). Since these patients have detailed presurgical cognitive assessment, this platform provides a unique ante-mortem window into understanding the altered cellular features that are associated with human cognitive decline (Ammothumkandy et al., 2025b). Our previous research in MTLE patients revealed the persistent presence of a pathological Dcx + immature astroglial cell in the epileptic hippocampus that was undetectable in control postmortem tissue (Ammothumkandy et al., 2022). The number of Dcx + immature astroglial cells in MTLE hippocampus trended toward an increase with age; however, the significance of this phenotype and its association with cognitive decline was not investigated. In this study, we first identified cognitive functions that strongly undergo an age-associated decline in epilepsy patients. We then assessed the significance of an age-associated increase in Dcx + immature astroglia and how it is associated with various cognitive functions. Our study identified an association between aging, a decline in fluid intelligence and an increase of immature astroglia in epilepsy patients. These findings highlight immature astroglia as an epilepsy-associated cellular feature corresponding to age-related cognitive decline and provides a basis for future mechanistic and translational studies.

## Methods and material

### Human participants

The cognitive study was approved by University of Southern California Institutional Review Board approval number: HS-10-00162, and Rancho Los Amigos National Rehabilitation Center and the Rancho Research Institute’s Institutional (RRI) Review Board, approval number: RRI IRB # 195 with proper data use agreement. We have complied with all relevant ethical regulations. Written consents were obtained from the patients to study clinical and demographic information. De-identified patient clinical details (age and disease duration of epilepsy, age of disease onset, sex, left vs. right hemisphere of effected hippocampus, education years) are provided in Supplementary Table S2. For the study of surgically resected epilepsy tissue, written consents for the surgical treatment of MTLE, tissue donation for the study as well as relevant clinical and demographic information collection (University of Southern California Institutional Review Board approval number: HS-17-00370) were obtained from all the patients prior to the surgery. Human hippocampal tissue was obtained from 29 patients undergoing an *en bloc* resection of their ipsilateral hippocampus for the treatment of medically resistant MTLE, following strict ethical guidelines. Both male and female cases were included to examine sex as a biological variable and are annotated in Supplementary Figures S1–S2d–i. Sex-based analyses were not performed due to limited sample size.

### Inclusion/exclusion criteria

The composition of each analysis was chosen based on data availability:

For Figure 1 (Cognition Data available in Spanish), we included (N = 40) MTLE patients who underwent en bloc hippocampal resection and had presurgical neuropsychological testing performed in Spanish, with available cognitive data.

**Figure 1:**
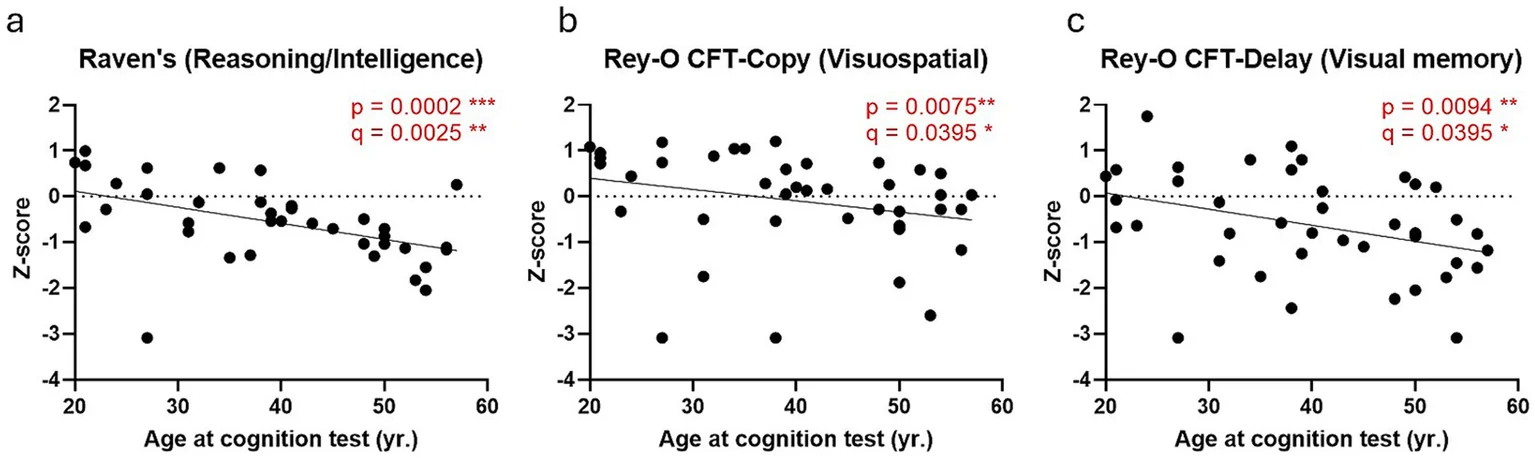
Age-associated cognitive decline in intelligence and visuospatial reconstruction and memory in epilepsy patients. Association between Age at cognitive test and cognitive z-scores calculated by two-tailed Spearman’s correlation for NeSBHIS tests: **(a)** Raven’s Intelligence, **(b)** Rey-O-CFT Copy visuospatial, **(c)** Rey-O-CFT Delay visuospatial memory, *p* values for Spearman’s correlation, q value for BH - FDR test for 15 cognitive tests, * *p*, q ≤ 0.05, ** *p*, q ≤ 0.01, *** *p*, q ≤ 0.001.

For Figures 2a–c (IHC Data available), we included (*N* = 29) MTLE patients who underwent en bloc hippocampal resection and had histological analysis available. Tissue was not available for research purposes in all cases (Figure 1). Immunohistochemistry quality was assessed qualitatively based on abundant presence of Dcx + stellar cells (Ammothumkandy et al., 2022). Neuropsychological data was not a criterion for inclusion in these analyses; therefore, participants who lacked appropriate cognitive data were also included (*N* = 10 of 29 samples).

**Figure 2:**
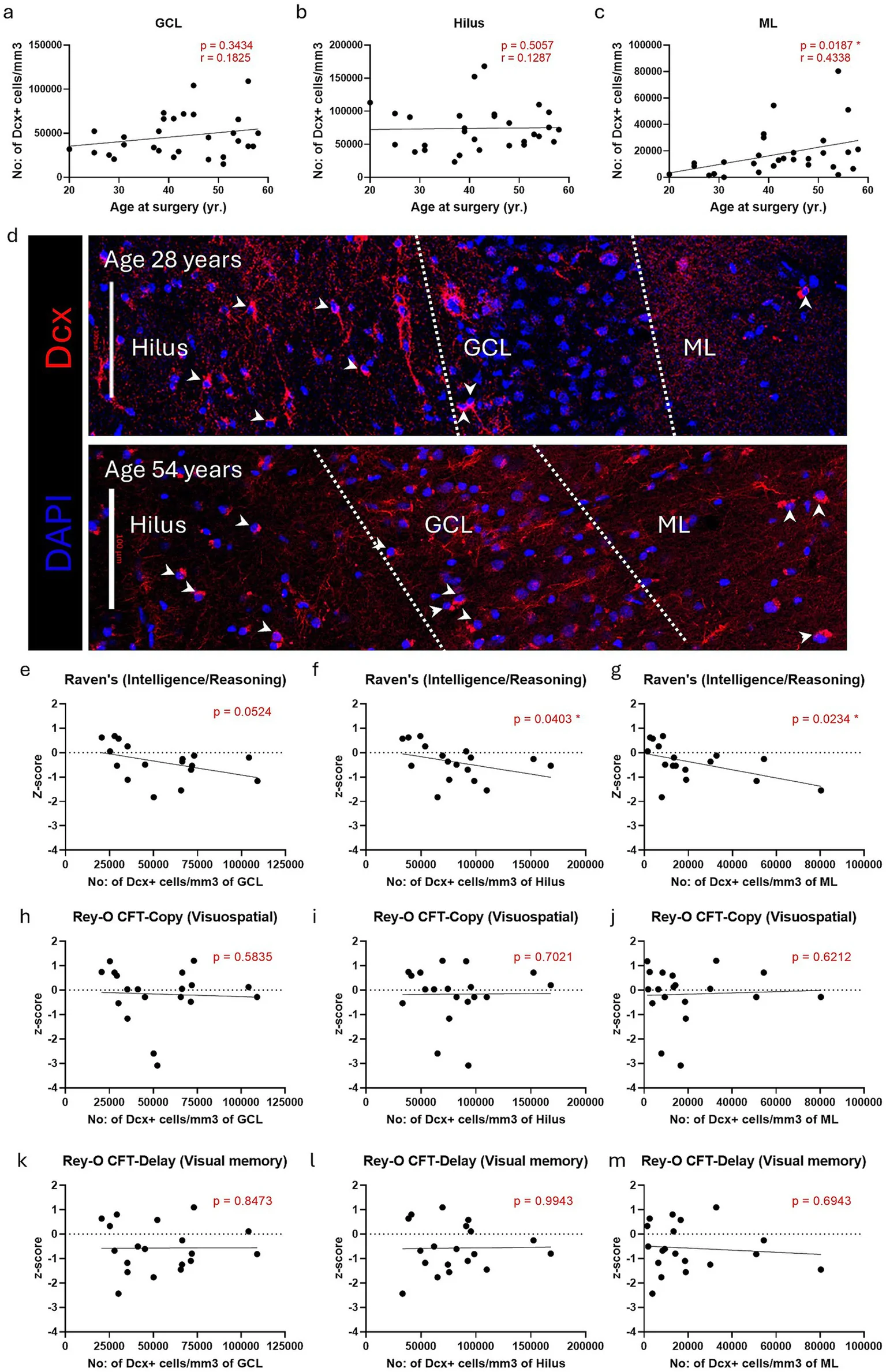
Immature astroglia associates with aging and a decline in fluid intelligence. **(a–c)** Association between Age at surgery and Number of Dcx + stellar immature astroglial cells/mm^3^ in the dentate gyrus **(a)** GCL, **(b)** hilus and **(c)** ML of *N* = 29 adult human MTLE patients. *p* values for Spearman’s correlation, * *p* ≤ 0.05. **(d)** Representative image for Dcx + stellar immature astroglial cells (red, marked by arrow) in the GCL (outlined by dotted lines), hilus and ML of the DG in a younger (Age: 28 years) and older (Age: 54 years) MTLE patient; Scale bar 100 μm. **(e–g)** Two-tailed Spearman’s Correlation between cognitive z-scores for Raven’s intelligence test and the number of Dcx + stellar immature astroglial cells/mm3 detected in the **(e)** GCL, **(f)** hilus, **(g)** and ML from the MTLE surgically resected hippocampus. *p* values for Spearman’s correlation, * *p* ≤ 0.05. **(h–j)** Two-tailed Spearman’s Correlation between cognitive z-scores for Rey-O-CFT Copy visuospatial test and the number of Dcx + stellar immature astroglial cells/mm3 detected in the **(h)** GCL, **(i)** hilus, **(j)** and ML from the MTLE surgically resected hippocampus. *p* values for Spearman’s correlation are not significant. **(k–m)** Two-tailed Spearman’s Correlation between cognitive z-scores for Rey-O-CFT Delay visuospatial memory and the number of Dcx + stellar immature astroglial cells/mm3 detected in the **(k)** GCL, **(l)** hilus, **(m)** and ML from the MTLE surgically resected hippocampus. *p* values for Spearman’s correlation are not significant.

For Figures 2e–m (Both IHC and Cognition data), we included only cases with both Spanish-language cognitive testing and available tissue for histological analysis (*N* = 19) to assess the association between immature astroglia and cognition.

### Cognitive assessment

Retrospective cognitive analysis of presurgical neuropsychological exams administered to Spanish-speaking patients (*N* = 40) with intractable TLE was performed as explained in our previous study (Ammothumkandy et al., 2025b). The Neuropsychological Screening Battery for Hispanics (NeSBHIS) was part of a comprehensive presurgical evaluation. Intelligence, attention, processing speed, language, visuospatial, visual memory, verbal learning and memory, executive functioning and fine motor dexterity were measured to assess epilepsy comorbidities (Supplementary Table S1). To account for variability in education, which influences neuropsychological test performance (Smith et al., 2020), subtest raw scores were converted to z-scores using education-based normative data (Pontón et al., 1996). Grooved pegboard was used instead of the fine-motor Pin test from NeSBHIS, which was normalized using age and education based normative data as in Smith, et.al, 2020 (Smith et al., 2020). Upper and lower cut off values for z-scores were set at +/− 3.09 to eliminate potential outliers due to very superior performance (z-scores > + 3.09) and clinically profound impairment (<3.09).

### Human MTLE hippocampal tissue processing

The surgery (*N* = 29) was performed by neurosurgeons at the Keck Medical Center or Los Angeles General Medical Center. For the cases where association between immature astroglia and cognition was analyzed (*N* = 19), the surgery was performed within a year of neuropsychology test for 10 out of 19 patients. The rest of the cases underwent surgery within 5 years of test (*N* = 4 after 1 year, *N* = 1 after 2 years, *N* = 2 after 3 years, *N* = 2 after 4 years). Surgically resected hippocampal tissue was transported to the laboratory within 30 min to 1 h of surgical removal in a tube containing HypoThermosol^®^ FRS (BioLife Solutions, Bothell, WA) solution maintained at approximately 4 °C in an ice box.

Hippocampal tissue was cut into 400 μm thick slices in chilled (4 °C), oxygenated (95% O_2_, 5% CO_2_) N-methyl-D-glycamine - artificial cerebrospinal fluid (aCSF) solution (NMDG-aCSF; NMDG 93 mM; KCl 2.5 mM; NaH_2_PO_4_ 1.2 mM; NaHCO_3_ 30 mM; HEPES 20 mM; Glucose 25 mM; Sodium ascorbate 5 mM; Thiourea 2 mM; Sodium pyruvate 3 mM; MgSO_4_ 10 mM; CaCl_2_ 0.5 mM) using a vibrating microtome (Leica VT1200S, Leica Biosystems, Germany). Vertical deflection of the blade was minimized with Vibrocheck technology (Leica Biosystems, Germany). Slicing parameters were set at speed 0.15–0.5 mm/s and vibration amplitude 1.5 mm. Hippocampal slices were first used for multi electrode array (MEA) experiments explained in our previous study (Ammothumkandy et al., 2022), where we assessed the relationship between MEA-defined neuronal hyperactivity and Dcx + immature astroglia. Slices were allowed to recover for a minimum of 1 h in a chamber with oxygenated (95% O_2_, 5% CO_2,_ 32 °C) aCSF using one of the following formulations (Formulation 1: NaCl 124 mM; KCl 4 mM; NaHCO_3_ 26 mM; Glucose 10 mM; CaCl_2_ 2 mM; MgCl_2_ 2 mM solution, Formulation 2: NaCl 124 mM; KCl 2.5 mM; NaH_2_PO_4_ 1.2 mM, NaHCO_3_ 24 mM; HEPES 5 mM, Glucose 12.5 mM; CaCl_2._2H_2_O 2 mM; MgSO_4_.7H_2_O 2 mM) before MEA recording. Inter*-*ictal activity in the hippocampal slice was recorded using MEA1060-Inv extracellular multi-electrode array recording system and MC Rack v3.9.1 software (Multi Channel Systems, Germany). Pictures of the slice relative to the recording electrode location were acquired on an inverted microscope (Leica DM-IRB, Germany) with a digital image capture system (Spot RT Digital Camera, Spot Imaging, Sterling Heights, MI) and Leica Application Suite software (Leica Microsystems, Buffalo Grove, IL). Continuous oxygenated, warm (32 °C) aCSF at a rate of 6 mL/min was circulated in the MEA ring well during tissue recording. A 5 min baseline recording was performed in aCSF followed by a 20–30 min recording in a modified aCSF solution containing high (8 mM) potassium, low (0.25 mM) magnesium and 100 μM 4-aminopyridine (4-AP) to induce inter-ictal like activity. Detailed MEA method is explained in our previous study identifying immature astroglia in human MTLE (Ammothumkandy et al., 2022). Following the MEA recording, slices were immediately fixed using 4% PFA for immunohistochemistry.

### Immunohistochemistry and cell quantification

Slices were pre-treated in a similar manner for methodological consistency between cases. Slices were fixed in 4% PFA - made freshly from 16% PFA stored at −20 °C - for 30 min at room temperature and washed thoroughly with 1x phosphate buffered saline (PBS) overnight. The slices were dehydrated using 30% sucrose in PBS at 4 °C. After 1 week, once the slice sank it was embedded in O. C. T compound (Tissue-Tek), ensuring the Dentate Gyrus (DG) region is flat. Slices were further sub-sectioned by cryostat (Leica CM 3050 S) into 30 μm and mounted to super frost plus slides. The slides were dried overnight at room temperature and transferred to −20 °C for long term storage. Before performing immunohistochemistry, slides were equilibrated to room temperature for 2 h or at 37 °C for 30 min. Slides were further washed in PBS for 1 h, followed by blocking and permeabilization in 3% Bovine serum albumin (BSA), 10% Donkey serum in 0.1% Triton-X in PBS for 2 h at room temperature. The sections were incubated with Dcx primary antibody [Santa Cruz, sc-390645, dilution 1 in 33] for 18–20 h at 4 °C in 0.3% BSA, 1% Donkey serum and 0.1% Triton X in PBS. Following a 1-h equilibration to room temperature, slides were washed in 0.1% Triton X in PBS for 15 min 3 times. Secondary antibodies from Jackson Immuno research laboratories (Anti-mouse Alexa Fluor 488 (Cat No: 715–546-151) or Anti-mouse Cy3 [Cat No: 715–166-150] were diluted (1:200) from a stock solution of 0.6 mg/mL and incubated along with 1 μg/mL of DAPI (Roche, Cat No: 10236276001) for 2 h at room temperature in 0.3% BSA, 1% Donkey serum and 0.1% Triton X. Sections were washed 3 times in 0.1% Triton X in PBS for 15 min and mounted with mounting media prepared in the lab. Glycerol-based PVA -2.5% DABCO mounting media was prepared by first adding 4.8 g PVA (Sigma P8136) to 12 g glycerol, mixing well, and adding 12 mL distilled water. After overnight mixing in rotor, 24 mL 0.2 M Tris–HCL pH 8–8.5 was added, and the tube was heated to 50 °C for 10–30 min in water bath with periodic mixing until the mixture was completely dissolved. The tube was further centrifuged at 5000 g for 15 min and the supernatant was collected. 1.25 g DABCO (Sigma D2522) was added to 50 mL supernatant, heated to 50 °C for 10–30 min and then centrifuged at 5000 g for 15 min to collect the supernatant. The supernatant mounting media mix was aliquoted and stored at −20 °C for long term storage and thawed at room temperature for mounting the tissue. After mounting media solidifies between the slide and cover glass whole DG region was imaged at 20X using a Zeiss LSM 700 confocal microscope automated tile scan. All cell quantification was performed manually in Zeiss blue software. Granular cell layer (GCL) boundaries were marked using the DAPI channel around the seemingly compact cell layer leaving a gap of two cell nuclear distance on both sides of the hilus and molecular layer. Dcx + cells with astroglial stellar morphology were analyzed in representative sub regions of the GCL. An adjacent region of hilus and molecular layer (ML) spanning 300 μm and 150 μm, respectively, was also marked and analyzed. In our previous study on human MTLE, we found that all Dcx^+^ stellar cells co-expressed GFAP and S100β, consistent with an immature astroglial phenotype, while lacking co-expression with markers of granule neurons (Prox1^+^), mossy cells (GluR2/3^+^), interneurons (Gad65/67^+^), microglia (Iba1^+^), and immature neurons (PSA-NCAM^+^) (Ammothumkandy et al., 2022). In the present study, Dcx + cells with stellar morphology were used as a practical index of immature astroglia. Dcx + immature astroglia were quantified for *N* = 29 cases (Figures 2a–c), of which *N* = 17 samples were derived from our previous study (Ammothumkandy et al., 2022). An additional *N* = 12 samples were subsequently processed using the same staining and quantification pipeline that minimizes batch effects and inter-run variability (Supplementary Figures S2a–c).

### Quantification and statistical analysis

All the statistical analysis was performed using GraphPad Prism 11. No statistical methods were used to predetermine sample size. No data points were excluded when available. Investigators were blinded to experimental parameters and group allocations. Individual data points are shown in all the graphs. A two-tailed Spearman’s correlation was performed to calculate the strength of linear relationship between two variables. The distribution of each continuous variable was first assessed for normality using the Shapiro–Wilk test. Normality results were mixed across the cognitive data points in Figure 1 and Figures 2e–m. Due to substantial variability in the human samples, non-parametric Spearman correlation was used for these analyses as the more appropriate and conservative approach. For Figures 2a–c, the Shapiro–Wilk test indicated a significant deviation from normality, and therefore Spearman’s correlation was the most appropriate. The Spearman’s correlation analysis was followed by a multiple test adjustment for the number of correlation tests compared using Benjamini and Hochberg Original False Discovery Rate (BH-FDR) method. The N value, correlation coefficient, *p*-value and q-value for these analyses are reported. Multiple linear regressions were performed using the least-squares method to evaluate the independent association of each predictor with the outcome while adjusting for other covariates. Overall model *R*^2^, beta, 95% confidence intervals, *p*-values, and the partial *R*^2^ values are reported for each predictor. Partial *R*^2^ was calculated for each predictor: Partial *R*^2^ = t^2^ / (t^2^ + residual degrees of freedom) to quantify its unique contribution to cognitive performance after adjustment for the other covariate.

## Results

### Cognitive performance in intelligence and visuospatial memory declines with age in epilepsy patients

Presurgical cognitive assessment of pharmacoresistant MTLE patients was performed using the Neuropsychological Screening Battery for Hispanics (NeSBHIS) (Pontón et al., 1996; Smith et al., 2020) and Grooved pegboard fine motor test (Supplementary Table S1). We then determined the cognitive domains that declined significantly with age at cognitive test. NeSBHIS z-scores were normalized for education, and Grooved Pegboard fine motor test (Smith et al., 2020) was normalized for age and education. Cognition was assessed in *N* = 40 MTLE patients who underwent cognitive tests in Spanish, with an average age (*M* = 39.88 years; SD = 11.56), disease duration (*M* = 26.74 years; SD = 14.05) and reported years of education (*M* = 7.9 years; SD = 3.51; Supplementary Table S2). The strength of the correlation between age at cognitive test and cognitive performance was determined by Spearman’s correlation followed by multiple test adjustment using Benjamini and Hochberg Original False Discovery Rate (BH-FDR) method (Supplementary Table S3). The strongest age associated declines were observed for the domains of 1) fluid intelligence and reasoning (Figure 1a), (2) visuospatial reconstruction (Figure 1b), and (3) visuospatial memory (Figure 1c). Other domains that significantly declined with age as shown by Spearman’s test but did not withstand multiple corrections with BH-FDR test were (1) Verbal short delay recall, (2) visual attention and processing speed, (3) Processing speed and graphomotor speed, (4) Executive functioning and divided attention (Supplementary Table S3; Supplementary Figure S1). In summary, this cohort of Spanish-speaking MTLE patients demonstrate age-associated cognitive decline most significantly in fluid intelligence and visuospatial reconstruction and memory domains.

Since fluid intelligence and visuospatial reconstruction and memory are domains that also decline with disease duration (Ammothumkandy et al., 2025b), we performed multiple linear regression analyses to determine whether age remained independently associated with these cognitive domains (Supplementary Table S5). After adjustment, neither age nor disease duration were independent predictors of fluid intelligence, visuospatial reconstruction, or visuospatial memory. For fluid intelligence, however, the model including age and disease duration was significant (*p* = 0.0065). Age showed a stronger inverse association with fluid intelligence performance than disease duration, as reflected by its larger effect estimate and lower *p* value (age: beta = −0.0256, *p* = 0.0838; disease duration: beta = −0.0114, *p* = 0.3326). Age accounted for 8.3% of the residual variance in fluid intelligence performance after adjustment for disease duration (partial *R*^2^ = 0.083), compared with 2.7% accounted for by disease duration after adjustment for age (partial *R*^2^ = 0.027). Collectively, these findings suggest that age may have a stronger adjusted association with fluid intelligence than disease duration.

### An increase in immature astroglia associates with aging and a decline in fluid intelligence

We next investigated whether age-associated cognitive decline in MTLE patients was linked to a pathological Dcx + stellar immature astroglial cell that we had previously identified in surgical epileptic hippocampus resections (Ammothumkandy et al., 2022). In that study, we observed that they co-expressed canonical astroglial markers GFAP and S100β, while also lacking marker co-expression of granule neurons (Prox1^+^), mossy cells (GluR2/3^+^), interneurons (Gad65/67^+^), microglia (Iba1^+^), and immature neurons (PSA-NCAM^+^). The Dcx + stellar immature astroglia were absent in control postmortem hippocampus confirming their pathological identity (Ammothumkandy et al., 2022). The previous study was performed in *N* = 17 MTLE cases that showed an increasing trend of number of Dcx + immature astroglial cells with age at surgery. In the current study, we included 12 additional MTLE cases for a combined N = 29 MTLE cases and then determined the association between Dcx + immature astroglia numbers and age at surgery. Dcx + stellar immature astroglial cells showed a significant (*p* = 0.0187) age- associated increase in the molecular layer (ML) of the dentate gyrus (DG), whereas in the granular cell layer (GCL) and hilus the levels were persistent with no age-associated changes (Figures 2a–d). In both the prior (Ammothumkandy et al., 2022) and current larger cohort (*N* = 29), age and disease duration exhibited a positive correlation (*r* = 0.4093, *p* = 0.0275) (Supplementary Figure S3a). However, the number of Dcx + immature astroglia in GCL, Hilus and ML did not display an association with disease duration (Supplementary Figures S3b–d). We also performed multiple linear regressions to determine whether Dcx + immature astroglia remained independently associated with age after adjusting for disease duration (Supplementary Table S6). Overall model fit trended toward significance (*p* = 0.08) in the ML and was not significant in the GCL and hilus. In the ML, age at surgery was a significant positive predictor of Dcx + immature astroglia counts (*p* = 0.0275), uniquely explaining 17.3% of the variance (partial *R*^2^ = 0.173); whereas disease duration was not significant and explained only 2.7% of the variance (partial *R*^2^ = 0.027) of Dcx + immature astroglia in ML. Both age at surgery and disease duration did not significantly predict Dcx + immature astroglia in the GCL or hilus. Similar to the spearman’s correlation analysis (Figures 2a–c), multiple linear regression analyses support a region-specific positive association between age at surgery and Dcx + immature astroglia in the ML (Supplementary Table S6).

Next, we assessed whether a greater number of Dcx + immature astroglial cells correlated with a decline of fluid intelligence, visuospatial reconstruction and memory domains in N = 19 Spanish MTLE cases for which we had both histology and cognition data available from the same individuals. Spearman’s correlation test showed a statistically significant negative association between fluid intelligence z-scores and the number of Dcx + stellar immature astroglial cells in the hilus (*p* = 0.04) and ML (*p* = 0. 02; Figures 2f,g). In the GCL, the association was trending toward significance (*p* = 0.052; Figure 2e). However, the correlations did not withstand the BH-FDR multiple test adjustment, likely due to a weaker association. Visuospatial reconstruction and memory (Figures 2h–m), and other cognitive domains also showed a lack of significant spearman’s correlation with the number of Dcx + immature astroglial cells in the GCL, hilus and ML (Supplementary Table S4). Therefore, an age-related decline in fluid intelligence and reasoning is associated with an increase in pathological immature astroglia numbers in epilepsy patients.

We next used multivariable linear regression to assess whether age and disease duration, along with Dcx + immature astroglia counts in GCL, Hilus or ML jointly predicted Raven’s test performance (Supplementary Table S7). The overall models were significant, and age showed a trend toward significance (*p* = 0.055 in Hilus, *p* = 0.08 in ML) in predicting fluid intelligence, while none of the individual predictors remained independently significant when modeled together. Age at surgery accounted for the largest unique proportion of residual variance in fluid intelligence (partial *R*^2^ = 12.6–25.4%), followed by Dcx immature astroglia measures (8.3–15.6%), whereas disease duration accounted for little unique variance (3.3–4.1%). We then removed either age or disease duration from the model. In models including only age along with Dcx + immature astroglia counts in GCL, Hilus or ML as predictors, age was consistently negatively associated with fluid intelligence across the GCL, hilus, and ML, whereas Dcx + immature astroglia counts were not significant. In models including only disease duration along with Dcx + immature astroglia counts in GCL, Hilus or ML as predictors, both Dcx + immature astroglia counts and disease duration were significantly and negatively associated with fluid intelligence in all three hippocampal regions. Together, these findings suggest that Dcx + immature astroglia are associated with fluid intelligence in disease-duration-adjusted models, but this relationship is attenuated after accounting for age at surgery, consistent with shared variance between Dcx + immature astroglia and age in relation to cognitive performance.

## Discussion

Astroglial dysfunction is increasingly recognized as a driver of cognitive deterioration in rodent models (Habbas et al., 2015; Santello et al., 2019), but its contribution to human cognitive decline has remained undetermined. In this study, we combined histological and neuropsychological analyses in epilepsy patients to reveal age-related intelligence decline correlated with the increased presence of pathological immature astroglia. Although we observed a pronounced age-associated decline in visuospatial reconstruction and visuospatial memory, these domains were not associated with immature astroglia levels. Our observations suggest an association between immature astroglia, aging and cognitive decline in epilepsy patients.

The significance of glial cells to human cognition was first highlighted by the observation of a higher ratio of glial cells to neurons in the brain of Albert Einstein, an extraordinarily intelligent scientist with exceptional visuospatial and mathematical reasoning skills, compared to control individuals (Diamond et al., 1985; Falk et al., 2012). Furthermore, transplantation of human embryonic glial progenitor cells into mice formed functional mature astrocytes that improved mouse learning and memory abilities, providing causal evidence that human astroglia can actively modulate cognitive function *in vivo*(Han et al., 2013). However, a direct cellular association between human astroglia and specific cognitive functions in humans remains largely unexplored. In this study, the analysis of 15 cognitive tests enabled us to identify a precise association between higher numbers of pathological immature astroglia and a decline in the reasoning and fluid intelligence domain. Our study implicates astroglia in human cognition, providing a potential cellular target to reverse age-associated cognitive decline, particularly within the fluid intelligence domain in epilepsy patients. In our previous work, we identified a positive correlation between immature neurons and verbal learning in MTLE patients (Ammothumkandy et al., 2025b). As cell type–specific combination therapies targeting both neurons and astroglia for cognitive disorders emerge (Li et al., 2025), our findings underscore astroglia as a promising treatment target for patients experiencing intelligence decline, whereas boosting immature neurons may preferentially enhance verbal learning.

In humans, evidence for the role of astroglia in cognition is largely indirect and comes from markers of astroglial reactivity, which have mainly been associated with global cognitive status rather than specific domains (Lye et al., 2019; Wu et al., 2023; Peretti et al., 2024). Experimental work in rodents provides mechanistic support for these observations, showing that astrocyte-specific manipulations can alter learning and memory performance (Santello et al., 2019). In our MTLE cohort, however, we did not observe a correlation between immature astroglia and decline in verbal or spatial learning and memory. These results may be due to our moderate sample size, which limits the sensitivity to reveal weaker associations. Instead, our study highlights the significance of investigating astroglial role toward fluid intelligence and reasoning, such as performing the Binary Tree Maze and the fast-mapping behavioral tasks that specifically access reasoning in mouse models (Matzel and Sauce, 2017). The association between pathological immature astroglia in hippocampus to intelligence decline was unexpected, because frontal regions are the most critical contributors to human fluid intelligence, as shown by lesion–deficit mapping studies (Cipolotti et al., 2023). However, the hippocampus can also potentially influence Raven’s fluid intelligence scores as it involves relating visual features and processing spatial layouts (Kumaran and Maguire, 2005; Hartley et al., 2007). In addition, hippocampal volume and fluid intelligence are linked in older, but not younger adults (Reuben et al., 2011). Further research is needed to determine whether age-related astroglial dysfunction in the hippocampus contributes to fluid intelligence impairments via disruption of hippocampal–frontal communication, as has been shown in relation to memory in young mice (Sardinha et al., 2017; Kol et al., 2020).

Astrocyte dysfunction is known to occur in rodent aging via an increase in neuroinflammatory reactive astrocytes and a decline in homeostatic functions (Clarke et al., 2018; Popov et al., 2021; Verkhratsky et al., 2022). Engrafting glial progenitor cells reverses decline of sensory function in aged mice, suggesting a functional role for younger astroglia in restoring cognition (Yang et al., 2022). Yet, an understanding about astroglial dysfunction in relation to human aging is very limited (Verkerke et al., 2021). Our study identifies an age-associated increase in immature astroglia in the ML of the hippocampal dentate gyrus of epilepsy patients. Disease associated astroglia are known to have immature status, impaired homeostatic functions and increases in pro-inflammatory signatures (Lattke and Guillemot, 2022). Therefore, the age-associated increase in immature astroglia observed in epilepsy patients might also contribute to cognitive dysfunctions by similar molecular mechanisms. Alternatively, a severely altered hippocampal niche with impaired cognition might lead to accumulation of immature astroglia. Future mechanistic studies are required to determine whether immature astroglia act as a cause or consequence of cognitive decline. Previous studies have shown accelerated brain aging and cognitive decline in human epilepsy patients (Sen et al., 2018). We provide a novel cellular association to astroglial maturation changes that occur with age in epilepsy patients, likely increasing their vulnerability to cognitive decline. In addition to astroglial cells, aberrant neural circuits, neuronal hyperactivity, blood–brain barrier leakage, oxidative stress, neuroinflammation, neurodegeneration might all collectively contribute to cognitive decline in MTLE (Ammothumkandy et al., 2025a). Immature astroglia may therefore act in concert with these processes rather than as an independent driver. Our study identifies an astroglial connection to human cognition, providing a potential cellular target to alter age-associated cognitive decline in epilepsy patients. Further research is needed to understand if immature astroglia causally contributes to intelligence decline and whether similar shifts in astroglia maturation state occur with normal aging and other age-associated cognitive disorders.

### Limitations

(1) Our study finds a negative association between immature astroglia prevalence and human fluid intelligence in MTLE patients; however, future mechanistic studies will be required to determine whether immature astroglia causally contributes to cognitive decline. (2) The small sample size in our study underscores the need for validation and extension in larger cohorts. For example, Gender-based analyses were not performed because of limited sample size. Similarly, the multivariable regression analyses have limited power to identify independent effects. However, the overall results from multiple linear regression analysis and Spearman correlation analyses are consistent, supporting associations among age, Dcx + immature astroglia, and Raven’s performance. (3) The potential relationship with additional clinical factors, such as seizure frequency, was also not examined due to lack of complete seizure records. Our previous research using MEA recordings found Dcx + immature astroglia activity is inversely correlated with interictal neuronal hyperactivity, with fewer immature astroglia present near hyperactive regions (Ammothumkandy et al., 2022). Therefore, Dcx + immature astroglia are well positioned to modulate neuronal hyper-activity and potentially influence or be influenced by seizure frequency. (4) The immature astroglial identity of the Dcx + population in the human MTLE hippocampus was established in our prior work (Ammothumkandy et al., 2022), in which all Dcx + cells co-expressed the astroglial markers S100β and GFAP. The use of additional astroglial markers and high-throughput molecular approaches, such as single-cell sequencing and spatial transcriptomics, can more comprehensively define the molecular identity of these Dcx + immature astroglia and signaling pathways that mediate cognitive decline.

## Data Availability

The original contributions presented in the study are included in the article/Supplementary material, further inquiries can be directed to the corresponding author/s.
