# Supplementary material for: An age-related decline in human fluid intelligence corresponds with immature astroglia prevalence
Source: Front Cell Neurosci. 2026 Oct 20;20:1926804. doi: 10.3389/fncel.2026.1926804 (PMC13727376; doi:10.3389/fncel.2026.1926804)
Supplement: Supplementary file 1 [file Supplementary_file_1.pdf]

Supplemental Material

Title: An age-related decline in human fluid intelligence corresponds with immature astroglia prevalence

Authors: Aswathy Ammothumkandy, Luis Corona, Nadia A. Atai, Aidin Abedi, Nora Jimenez, Michelle Armacost, Lina M. D'Orazio, Virginia Zuverza-Chavarria, Alisha Cayce, Mohammad Shariq, Ryan McGinn, Darrin Lee, Brian Lee, Christianne Heck, Jonathan J. Russin, Charles Y. Liu, Jason A.D. Smith and Michael A. Bonaguidi.

Contents:
Supplemental Tables S1 - 7
Supplemental Figures S1 - 3

Table S1

**Cognitive Domain**	**Test (Description)**
**Reasoning / Intelligence**	Raven's Standard Progressive Matrices<br><br>(Nonverbal/visual reasoning task to estimate fluid intelligence. Often regarded as culture fair measure.)
**Attention, Processing Speed and Executive Functioning**	Digit Symbol (Processing speed and graphomotor speed)<br><br>Color Trails 1 (Visual attention and processing speed)<br><br>Color-Trail 2 (Divided attention, cognitive flexibility, executive functioning)
**Language / Verbal Ability**	Pontón-Satz Boston Naming Test (P-S BNT; confrontational object naming drawn figures)<br><br>Controlled Oral Word Association Test (COWAT, FAS; measure of phonemic verbal fluency)<br><br>Digit Span (Verbal Attention and working memory, repetition of digits forward and then reverse)
**Verbal Learning and Memory**	World Health Organization – University of California, Los Angeles Auditory Verbal Learning Test (WHO-UCLA AVLT).<br><br>Trials 1-5 (Fifteen words presented over five trials to promote learning)<br><br>Trial 6 (Second 15-word list, an interference trial)<br><br>Trial 7 (Short delay recall of the original items, immediately follows Trial 6)<br><br>Trial 8 (20-minute, long delay recall)
**Visuospatial**	Block Design (Visuospatial constructional ability)<br><br>Rey-Osterrieth Complex Figure Test (ROCFT) -<br>Copy (Reconstruction of an intricate, two-dimensional geometric figure)
**Visual Memory**	ROCFT – Delay (10-minute delayed recall, reconstructing the ROCFT stimuli)
**Fine Motor Functioning**	Grooved Pegboard Dominant (GP-D) and Nondominant (GP-ND) hands<br><br>(task of manual dexterity, visuomotor integration and speed)

Table S1: Cognitive domains and the respective NeSBHIS (Neuropsychological Screening Battery for Hispanics) tests.

Table S2

Subject Number	Age of onset (yr.)	Age at Neuropsychology test (yr.)	Disease Duration at Neuropsychology test (yr.)	Age at Surgery (yr.)	Education (yr.)	Sex	Seizure Laterality	Data
MTLE 1	0	27	27	31	12	female	left	Cognition
MTLE 2	1	21	20	N.A.	11	male	right	Cognition
MTLE 3	1	23	22	24	6	male	right	Cognition
MTLE 4	1	53	52	53	12	male	left	Cognition, IHC
MTLE 5	1	57	56	57	12	male	right	Cognition, IHC
MTLE 6	1	N.A.	N.A.	25	N.A.	female	right	IHC
MTLE 7	3	31	28	32	9	male	left	Cognition
MTLE 8	3	56	53	56	3	female	right	Cognition, IHC
MTLE 9	4	52	48	N.A.	6	female	left	Cognition
MTLE 10	5	21	16	21	9	female	left	Cognition
MTLE 11	5	50	45	51	2	female	left	Cognition
MTLE 12	6	48	42	48	8	female	left	Cognition, IHC
MTLE 13	6	48	42	49	6	female	left	Cognition
MTLE 14	7	32	25	32	5	male	right	Cognition
MTLE 15	7	45	38	45	6	female	right	Cognition, IHC
MTLE 16	7	50	43	50	6	male	left	Cognition
MTLE 17	8	24	16	26	6	female	right	Cognition
MTLE 18	8	39	31	39	6	male	right	Cognition, IHC
MTLE 19	9	31	22	32	12	male	right	Cognition
MTLE 20	10	27	17	29	6	male	right	Cognition, IHC
MTLE 21	10	N.A.	N.A.	48	N.A.	male	right	IHC
MTLE 22	12	20	8	21	9	male	left	Cognition
MTLE 23	12	34	22	34	6	male	left	Cognition
MTLE 24	12	35	23	38	5	female	right	Cognition
MTLE 25	12	37	25	38	5	female	right	Cognition
MTLE 26	12	38	26	39	6	male	right	Cognition, IHC
MTLE 27	12	39	27	42	1	male	right	Cognition, IHC
MTLE 28	12	50	38	51	3	female	right	Cognition
MTLE 29	12	N.A.	N.A.	41	N.A.	female	left	IHC
MTLE 30	12	N.A.	N.A.	31	N.A.	male	right	IHC
MTLE 31	13	N.A.	N.A.	20	N.A.	female	right	IHC
MTLE 32	14	49	35	50	12	female	right	Cognition
MTLE 33	15	21	6	25	12	female	left	Cognition, IHC
MTLE 34	15	43	28	43	7	female	left	Cognition
MTLE 35	15	54	39	54	8	female	left	Cognition, IHC
MTLE 36	17	38	21	38	12	female	left	Cognition, IHC
MTLE 37	23	50	27	51	6	female	left	Cognition
MTLE 38	25	27	2	28	12	male	left	Cognition, IHC
MTLE 39	25	40	15	43	6	female	left	Cognition, IHC
MTLE 40	25	54	29	N.A.	14	female	right	Cognition
MTLE 41	28	54	26	54	14	female	right	Cognition, IHC
MTLE 42	28	N.A.	N.A.	31	N.A.	female	right	IHC
MTLE 43	30	38	8	38	12	male	left	Cognition, IHC
MTLE 44	30	N.A.	N.A.	37	N.A.	female	left	IHC
MTLE 45	30	N.A.	N.A.	51	N.A.	female	right	IHC
MTLE 46	33	41	8	41	12	female	right	Cognition, IHC
MTLE 47	34	41	7	45	3	female	right	Cognition, IHC
MTLE 48	47	N.A.	N.A.	51	N.A.	male	right	IHC
MTLE 49	49	56	7	56	8	female	right	Cognition, IHC
MTLE 50	52	N.A.	N.A.	58	N.A.	female	right	IHC

Table S2: Clinical, demographic information and experiments performed in MTLE cases (N = 50). MTLE - Mesial Temporal Lobe Epilepsy, IHC – Immunohistochemistry, N.A - Not Available. Disease duration, Age at surgery, Age of onset and Education in years (yr.).

Table S3

Cognitive Domain	Number of patients	Spearman's correlation coefficient	p-value	q-value
**Raven's**	38	-0.5674	0.0002***	0.0025**
**Rey-O-CFT Copy**	40	-0.4166	0.0075**	0.0395*
**Rey-O-CFT Delay**	40	-0.4055	0.0094**	0.0395*
**Block Design**	40	-0.3672	0.0197*	0.0533
**AVLT 7**	40	-0.3567	0.0239*	0.0533
**Color trails 1**	39	-0.3576	0.0254*	0.0533
**Digit Symbol**	40	-0.3306	0.0372*	0.0668
**Color trails 2**	38	-0.331	0.0424*	0.0668
**AVLT 8**	40	-0.2775	0.083	0.1162
**AVLT 5**	40	-0.2691	0.0932	0.1174
**Pegboard Non-dominant**	35	-0.267	0.121	0.1386
**Digit Span**	40	-0.2285	0.1561	0.1639
**Pegboard dominant**	36	-0.1571	0.3601	0.349
**P-BNT**	40	-0.04659	0.7753	0.6978
**COWAT/FAS**	37	-0.003742	0.9825	0.8253

Table S3: Two-tailed Spearman's Correlation between age and presurgical cognitive z-scores, followed by BH-FDR test for 15 cognitive tests* p, q ≤ 0.05, ** p, q ≤ 0.01, *** p, q ≤ 0.001.

Table S4

Cognitive Domain	Number of Patients	GCL		Hilus		ML	
		Spearman's correlation coefficient (r)	p-value	(r)	p-value	(r)	p-value
**Raven's**	17	-0.4807	0.0524	-0.5052	0.0403*	-0.5518	0.0234*
**Color trails 1**	19	-0.33	0.1677	-0.2615	0.2795	-0.4449	0.0563
**Pegboard dominant**	17	0.05302	0.8396	0.1208	0.6417	-0.4168	0.0969
**Digit Symbol**	19	0.337	0.1583	0.409	0.0821	0.3572	0.1333
**COWAT/FAS**	16	0.1708	0.5241	0.4389	0.0902	0.2342	0.3797
**AVLT 7**	19	-0.1021	0.6774	-0.01937	0.9373	-0.1796	0.462
**AVLT 8**	19	0.008858	0.9713	-0.04872	0.843	-0.147	0.548
**Color trails 2**	18	-0.2065	0.411	-0.1456	0.5643	-0.1435	0.5699
**P-BNT**	19	-0.03078	0.9004	-0.08004	0.7446	-0.1293	0.5978
**Rey-O-CFT Copy**	19	-0.1343	0.5835	-0.09394	0.7021	-0.1212	0.6212
**Rey-O-CFT Delay**	19	-0.04737	0.8473	-0.00175	0.9943	-0.09649	0.6943
**Digit Span**	19	0.1522	0.5339	-0.11	0.654	0.08095	0.7418
**AVLT 5**	19	0.1098	0.6546	0.1651	0.4993	-0.03953	0.8724
**Pegboard Non-dominant**	16	-0.04296	0.8748	0.2978	0.2604	-0.02963	0.9142
**Block Design**	19	-0.2476	0.3068	-0.1001	0.6835	0.002634	0.9915

Table S4: Two-tailed Spearman's Correlation between presurgical cognitive z-scores and the number of immature stellar Dcx+ astroglial cells/mm3 in the GCL, hilus, and ML of MTLE surgically resected hippocampus DG. p values for Spearman's correlation, * $p \leq 0.05$. Multiple test adjustment for 15 cognitive tests using BH-FDR test did not yield significant q values.

Table S5

Outcome	Overall model, p	R^2	Age β (95% CI), p	Disease duration β (95% CI), p	Partial R^2 for age	Partial R^2 for disease duration
**Raven's z-score**	F(2, 35) = 5.836, p = 0.0065**	0.2501	-0.02561 (-0.05483 to 0.003598), p = 0.0838	-0.01140 (-0.03495 to 0.01215), p = 0.3326	0.083 (8.3%)	0.027 (2.7%)
**Rey-O-CFT Copy z-score**	F(2, 37) = 2.281, p = 0.1164	0.1098	-0.007286 (-0.04703 to 0.03246), p = 0.7124	-0.02176 (-0.05439 to 0.01086), p = 0.1847	0.004 (0.4%)	0.047 (4.7%)
**Rey-O-CFT Delay z-score**	F(2, 37) = 2.903, p = 0.0674	0.1356	-0.02904 (-0.06879 to 0.01070), p = 0.1472	-0.007649 (-0.04028 to 0.02498), p = 0.6376	0.056 (5.6%)	0.006 (0.6%)

Table S5: Multiple linear regression models tested age and disease duration as predictors of Raven's z-score, Rey-O-CFT Copy z-score, and Rey-O-CFT Delayed Recall z-score.

Table S6

Outcome	Overall model, p	R^2	Age β (95% CI), p	Disease duration β (95% CI), p	Partial R^2 for age	Partial R^2 for disease duration
**Dcx+ cells in GCL**	F(2, 26) = 1.136, p = 0.3366	0.08034	703.3 (-264.0 to 1670), p = 0.1471	-278.2 (-934.9 to 378.6), p = 0.3919	0.079 (7.9%)	0.028 (2.8%)
**Dcx+ cells in Hilus**	F(2, 26) = 0.06718, p = 0.9352	0.005141	188.0 (-1233 to 1609), p = 0.7878	-161.7 (-1126 to 803.0), p = 0.7333	0.003 (0.3%)	0.005 (0.5%)
**Dcx+ cells in ML**	F(2, 26) = 2.765, p = 0.0815	0.1754	780.9 (93.57 to 1468), p = 0.0275*	-192.7 (-659.4 to 274.0), p = 0.4037	0.173 (17.3%)	0.027 (2.7%)

Table S6: Multiple linear regression models tested age and disease duration as predictors of Dcx+ immature astroglia in GCL, Hilus and ML.

Table S7

Predictors for the outcome – Raven's Z score	Overall model, p	R^2	Dcx^+ cells β (95% CI), p	Age β (95% CI), p	Disease duration β (95% CI), p	Partial R^2 for Dcx^+ cells	Partial R^2 for Age	Partial R^2 for Disease duration
**Dcx^+ cells in GCL + Age + Disease duration**	F(3, 13) = 5.606, p = 0.0109*	0.5640	−7.645e−06 (−2.290e−05 to 7.608e−06), p = 0.2986	−0.03355 (−0.08641 to 0.01931), p = 0.1935	−0.009566 (−0.03716 to 0.01803), p = 0.4672	0.083 (8.3%)	0.126 (12.6%)	0.041 (4.1%)
**Dcx^+ cells in Hilus + Age + Disease duration**	F(3, 13) = 6.224, p = 0.0075**	0.5895	−5.699e−06 (−1.429e−05 to 2.893e−06), p = 0.1755	−0.03992 (−0.08092 to 0.001086), p = 0.0555	−0.007573 (−0.03062 to 0.01547), p = 0.4903	0.136 (13.6%)	0.254 (25.4%)	0.037 (3.7%)
**Dcx^+ cells in ML + Age + Disease duration**	F(3, 13) = 6.464, p = 0.0065**	0.5987	−1.063e−05 (−2.546e−05 to 4.204e−06), p = 0.1456	−0.03629 (−0.07872 to 0.006148), p = 0.0876	−0.006813 (−0.02889 to 0.01526), p = 0.5165	0.156 (15.6%)	0.208 (20.8%)	0.033 (3.3%)
**Dcx^+ cells in GCL + Age**	F(2, 14) = 8.391, p = 0.0040**	0.5452	−4.270e−06 (−1.574e−05 to 7.203e−06), p = 0.4381	−0.04823 (−0.07915 to −0.01730), p = 0.0048**	—	0.044 (4.4%)	0.444 (44.4%)	—
**Dcx^+ cells in Hilus + Age**	F(2, 14) = 9.417, p = 0.0026**	0.5736	−4.436e−06 (−1.193e−05 to 3.057e−06), p = 0.2249	−0.04956 (−0.07749 to −0.02163), p = 0.0019**	—	0.103 (10.3%)	0.508 (50.8%)	—
**Dcx^+ cells in ML + Age**	F(2, 14) = 9.865, p = 0.0021**	0.5849	−8.870e−06 (−2.219e−05 to 4.454e−06), p = 0.1753	−0.04556 (−0.07472 to −0.01640), p = 0.0048**	—	0.127 (12.7%)	0.445 (44.5%)	—
**Dcx^+ cells in GCL + Disease duration**	F(2, 14) = 7.026, p = 0.0077**	0.5009	−1.441e−05 (−2.558e−05 to −3.246e−06), p = 0.0151*	—	−0.02360 (−0.04051 to −0.006683), p = 0.0097**	0.354 (35.4%)	—	0.390 (39.0%)
**Dcx^+ cells in Hilus + Disease duration**	F(2, 14) = 5.724, p = 0.0153*	0.4499	−9.437e−06 (−1.795e−05 to −9.234e−07), p = 0.0322*	—	−0.02362 (−0.04145 to −0.005793), p = 0.0131*	0.288 (28.8%)	—	0.366 (36.6%)
**Dcx^+ cells in ML + Disease duration**	F(2, 14) = 6.815, p = 0.0086**	0.4933	−1.721e−05 (−3.084e−05 to −3.584e−06), p = 0.0170*	—	−0.02018 (−0.03694 to −0.003417), p = 0.0217*	0.344 (34.4%)	—	0.323 (32.3%)

Table S7: Multiple linear regression models tested Dcx+ immature astroglia, age and disease duration as predictors of Raven's Z score.

Fig S1:

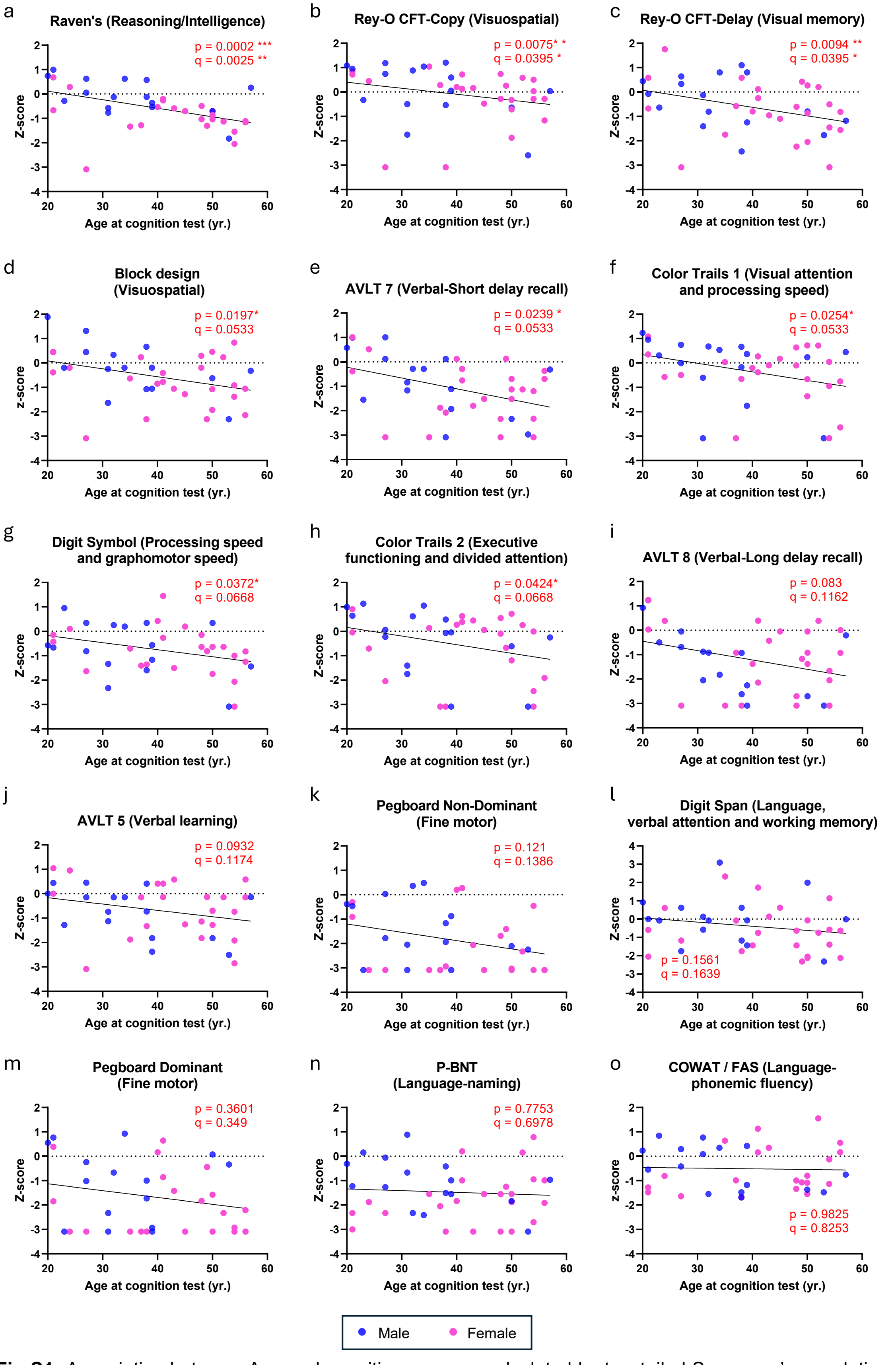

Fig S1: Association between Age and cognitive z-scores calculated by two-tailed Spearman's correlation, followed by multiple test adjustment using BH-FDR test for: a) Raven's Intelligence, b) Rey-O-CFT Copy visuospatial, c) Rey-O-CFT Delay visuospatial memory, d) Block Design visuospatial, e) AVLT 7 verbal short-delay recall, f) Color trails 1 visual attention, g) Digit Symbol processing speed, h) Color trails 2 executive functioning, i) AVLT 8 long-delay recall, j) AVLT 5 verbal learning, k) Pegboard Non-dominant fine motor control, l) Digit Span language attention, m) Pegboard dominant fine motor control, n) P-BNT language, o) COWAT/FAS language fluency. p values for Spearman's correlation, q value for BH - FDR test for 15 cognitive tests, * p, q ≤ 0.05, ** p, q ≤ 0.01, *** p, q ≤ 0.001. Female cases denoted in pink and Male cases denoted in blue.

Fig S2:

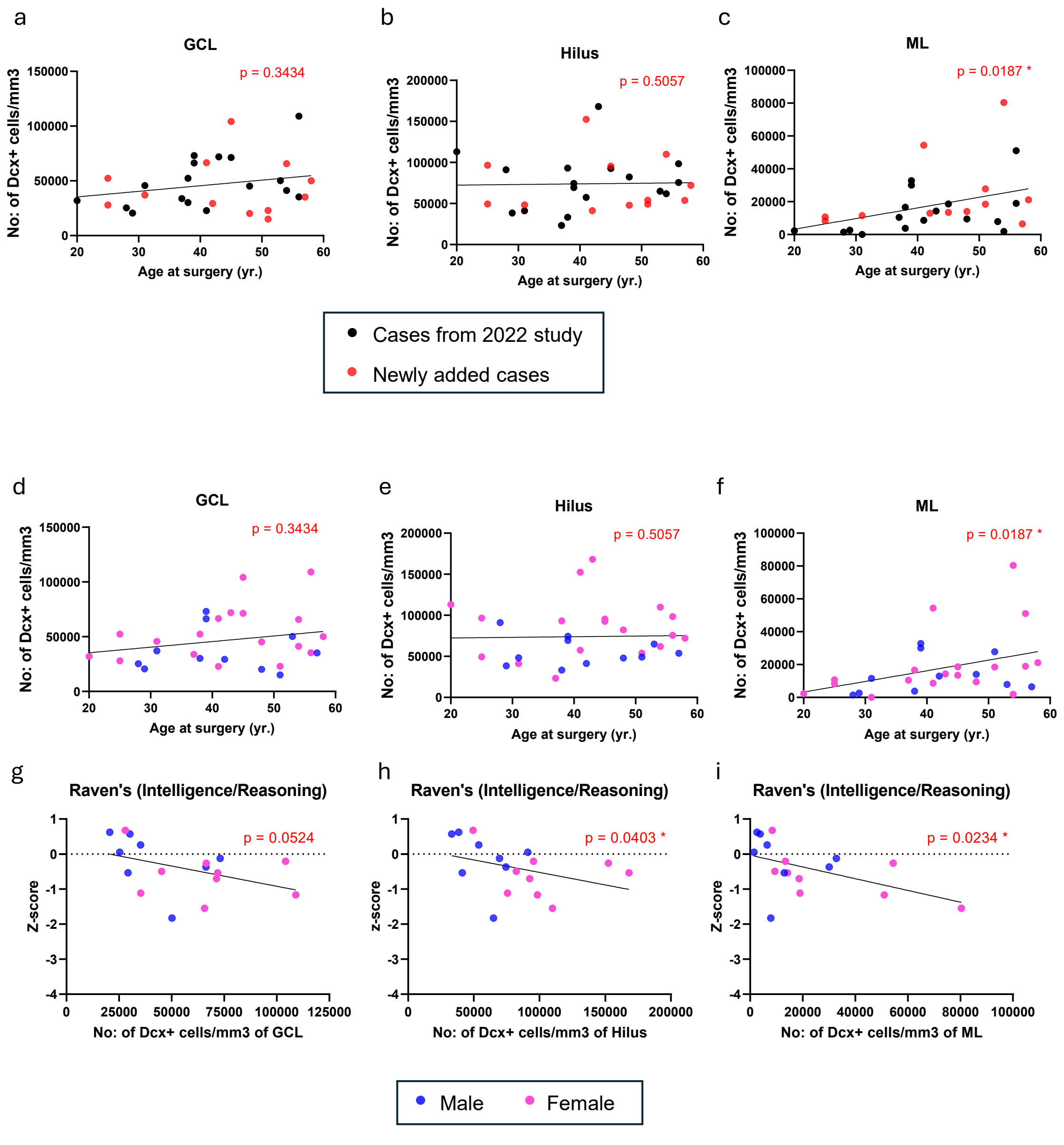

Fig S2: a-c) Association between Age at surgery and Number of Dcx+ stellar immature astroglial cells/mm3 in the dentate gyrus a) GCL, b) hilus and c) ML of N=29 adult human MTLE patients. Cases from Ammothumkandy et al 2022 study denoted in black (N =17) and newly added cases denoted in red (N = 12). d-f) Association between Age at surgery and Number of Dcx+ stellar immature astroglial cells/mm3 in the dentate gyrus d) GCL, e) hilus and f) ML of N=29 adult human MTLE patients. Female cases denoted in pink and Male cases denoted in blue. g-i) Association between cognitive z-scores for Raven's intelligence test and the number of Dcx+ stellar immature astroglial cells/mm3 detected in the g) GCL, h) hilus, i) and ML from the MTLE surgically resected hippocampus. Female cases denoted in pink and Male cases denoted in blue. p values for Spearman's correlation, * $p \leq 0.05$.

Fig S3:

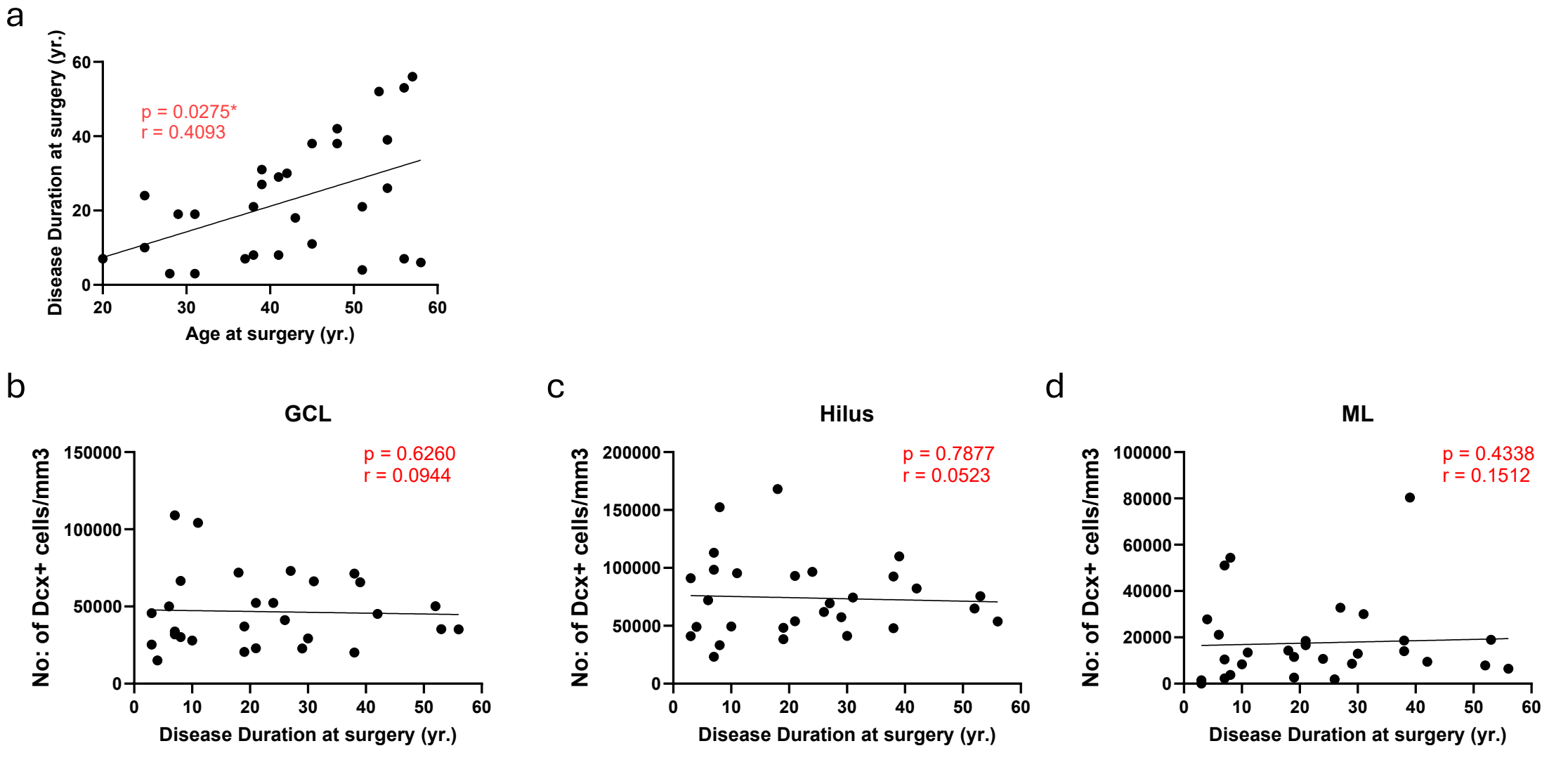

Fig S3: a) Association between Age and disease duration at surgery of N=29 adult human MTLE patients. b-d) Association between Disease duration at surgery and Number of Dcx+ stellar immature astroglial cells/mm3 in the dentate gyrus b) GCL, c) hilus and d) ML of N=29 adult human MTLE patients. p values for Spearman's correlation, * $p \leq 0.05$.
